# Analysis of spermidine’s effect on coronary heart disease risk using bidirectional Mendelian randomization and LC-MS/MS

**DOI:** 10.1186/s41065-025-00568-4

**Published:** 2025-09-26

**Authors:** Tianyi Wang, Li Lin, Yaodong Ding, Yang Zhang, Zehao Zhao, Ruixiang Feng, Yingxuan Bai, Zhennan Li, Yuncong Shi, Na Li, Yong Zeng

**Affiliations:** 1 Beijing Anzhen Hospital, Beijing Institute of Heart Lung and Blood Vessel Disease, Capital Medical University, Beijing, 100011 China; 2 Mass Spectrometry Research Institute, Beijing Gobroad Healthcare Group, Beijing, 100071 China

**Keywords:** Spermidine, Coronary heart disease, Mendelian randomization, LC-MS/MS, Cardiovascular biomarkers

## Abstract

**Background:**

This study examines the causal relationship between spermidine levels and coronary artery disease (CHD) risk using a bidirectional Mendelian Randomization (MR) approach.

**Methods:**

We employed genetic variants as instrumental variables to assess the influence of genetically predicted spermidine levels on CHD risk and vice versa. Data for the MR analysis were sourced from the UK Biobank and genome-wide association study datasets, focusing on single nucleotide polymorphisms (SNPs) associated with spermidine levels and CHD. The study also utilized liquid chromatography-tandem mass spectrometry (LC-MS/MS) for accurate quantification of spermidine in plasma samples.

**Results:**

Our analysis identified a significant association between lower genetically predicted spermidine levels and increased CHD risk. The LC-MS/MS results supported the accurate measurement of spermidine, highlighting its feasibility as a clinical biomarker.

**Conclusions:**

The findings suggest that reduced spermidine levels may be a significant risk factor for CHD. This study supports the potential of spermidine as a biomarker for CHD risk assessment and its development as a therapeutic target. The integration of genetic and biochemical methodologies enhances our understanding of the role of spermidine in cardiovascular health and its utility in managing CHD risk.

**Supplementary Information:**

The online version contains supplementary material available at 10.1186/s41065-025-00568-4.

## Introduction

Spermidine is a naturally occurring polyamine involved in a wide range of cellular processes, including cell growth, differentiation, and survival [1]. As a potent modulator of autophagy, spermidine plays a critical role in maintaining cellular homeostasis by promoting the degradation of damaged proteins and organelles, thus contributing to cellular repair and anti-aging effects [2, 3]. Additionally, spermidine has been shown to possess cardioprotective properties, helping to preserve endothelial function, reduce inflammation, and protect against oxidative stress [1, 4]—all key contributors to the development of cardiovascular diseases. Given these beneficial effects, spermidine’s role in cardiovascular health has garnered increasing attention.

Coronary heart disease (CHD), also known as ischemic heart disease, represents the clinical spectrum of disorders caused by impaired coronary blood flow resulting in myocardial ischemia [5]. This includes conditions such as myocardial infarction (MI), angina pectoris (both stable and unstable), silent myocardial ischemia, and sudden cardiac death. CHD is one of the leading causes of morbidity and mortality worldwide, accounting for a significant proportion of cardiovascular-related hospitalizations and deaths, especially in aging populations and developed countries [6]. The increasing burden of CHD imposes substantial socioeconomic costs and highlights the urgent need for effective preventive, diagnostic, and therapeutic strategies [7]. Well-established risk factors for CHD include hypertension, diabetes mellitus, dyslipidemia, smoking, obesity, sedentary lifestyle, and advancing age [8]. These factors accelerate atherosclerotic plaque formation, promote endothelial dysfunction, and increase the risk of acute coronary events through mechanisms involving inflammation and thrombosis [9].

Emerging evidence has identified a potential link between polyamine metabolism—particularly spermidine—and several of these traditional CHD risk factors [10]. Experimental and observational studies suggest that higher spermidine intake or circulating levels may reduce blood pressure, improve metabolic parameters such as insulin sensitivity, attenuate vascular aging, and protect against myocardial injury [11]. In addition, older adults, who are at greatest risk for CHD events, tend to exhibit lower endogenous spermidine levels, implicating this metabolite as a candidate for risk stratification and intervention. Thus, spermidine has garnered attention both as a biomarker for CHD risk and as a possible target for therapeutic development [12].

Mendelian Randomization (MR) is a powerful statistical method that has been instrumental in establishing causal relationships between modifiable exposures and health outcomes, particularly in the context of complex diseases and risk factors [13]. By utilizing genetic variants as instrumental variables, MR minimizes the biases introduced by confounding and reverse causality, making it an ideal approach for causal inference [14]. Traditionally applied in a unidirectional manner, recent advancements have extended MR to bidirectional analyses, allowing researchers to investigate the mutual influences between traits and exposures [15]. In the context of CHD, this approach can clarify whether altered spermidine levels contribute to CHD risk, or conversely, whether CHD and its treatments influence circulating spermidine concentrations.

In this study, we aim to elucidate the causal relationship between spermidine levels and CHD risk by employing a bidirectional Mendelian randomization (MR) framework. Furthermore, we utilize liquid chromatography-tandem mass spectrometry (LC-MS/MS) to accurately quantify spermidine concentrations in the plasma of patients with clinically defined CHD, as well as in healthy controls. Subgroup analyses are conducted to assess how spermidine relates to major CHD risk factors such as hypertension, hyperglycemia, and dyslipidemia. By integrating genetic epidemiology with advanced biochemical profiling, our research provides new insights into the potential of spermidine as a predictive biomarker and therapeutic target for CHD, and contributes to the broader understanding of metabolic influences on clinical coronary heart disease.

## Materials and methods

### Mendelian randomization study

#### Overview

**Fig. 1:**
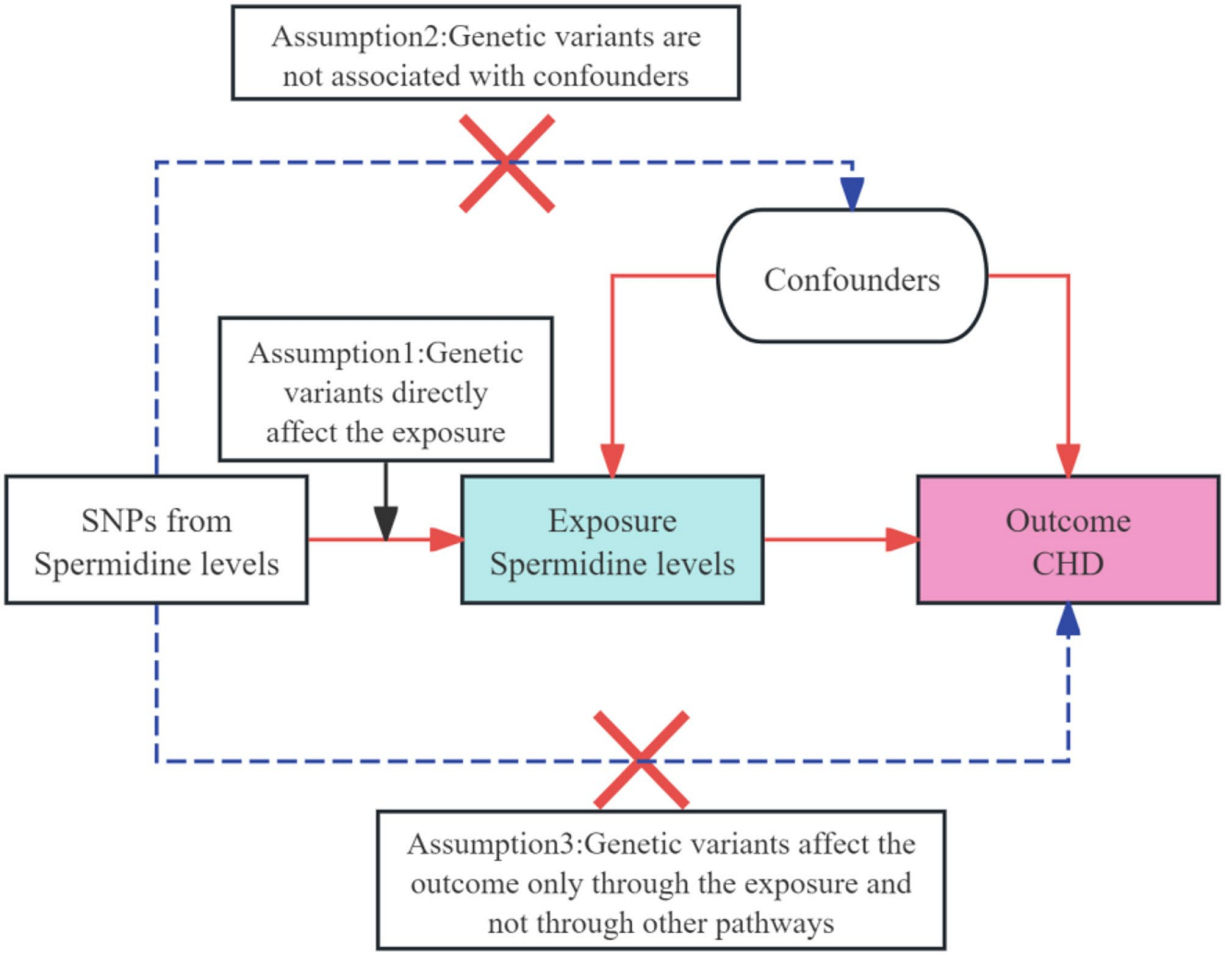
Schematics for bidirectional MR analysis of spermidine levels and CHD. All selected SNPs fulfill three basic assumptions. Single nucleotide polymorphisms, SNPs; Coronary heart disease, CHD

**Fig. 2:**
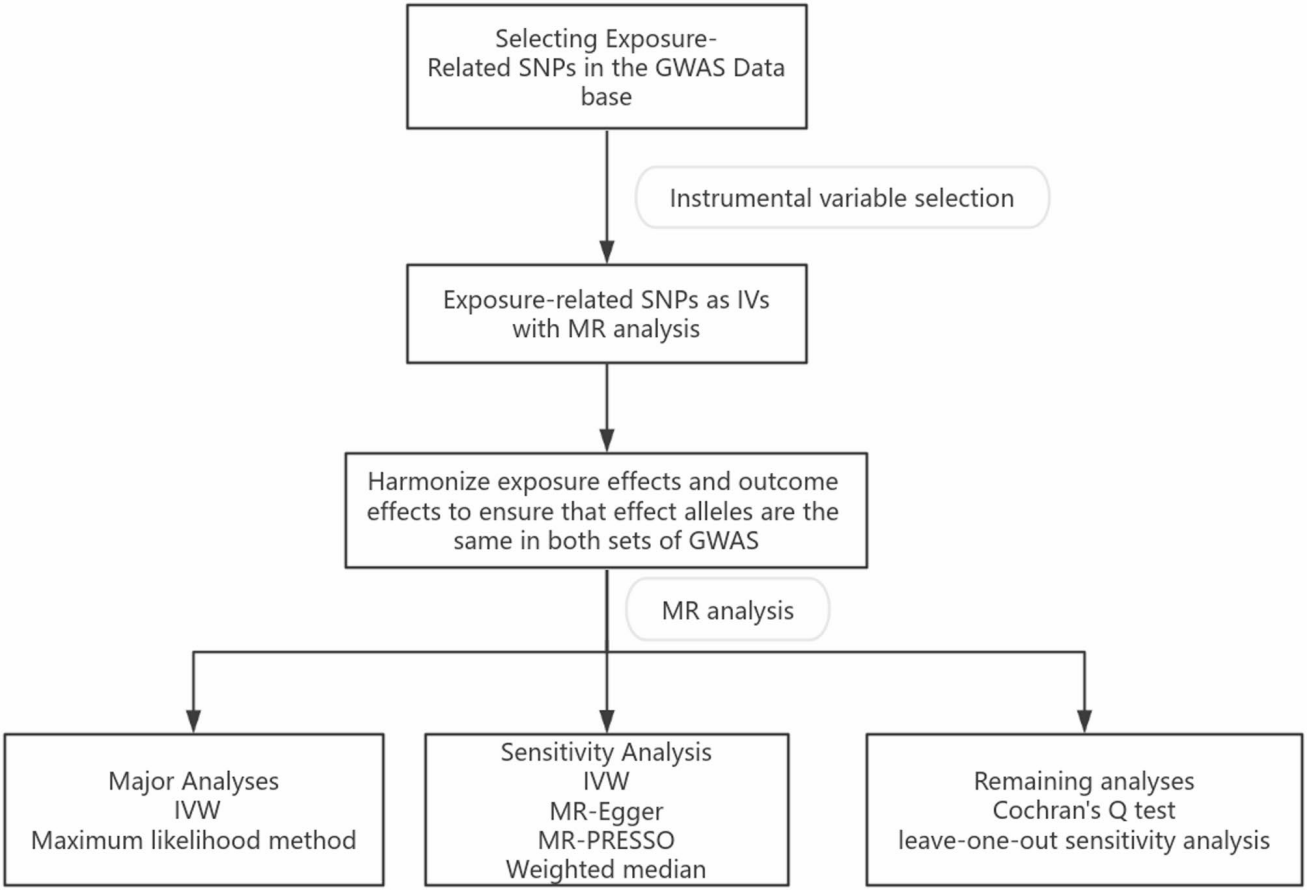
Flowchart of the analyses process. Inverse-variance weighted, IVW; MR pleiotropy residual sum and outlier, MR-PRESSO

This study employs a bidirectional two-sample MR approach to explore the causal relationship between plasma spermidine levels and CHD. Our analysis investigates both the influence of genetically predicted spermidine levels on CHD and vice versa. The robustness of the framework is ensured by adherence to the Strengthening the Reporting of Observational Studies in Epidemiology (STROBE) guidelines, adapted for MR studies (STROBE-MR) [16]. A schematic of our bidirectional MR analysis is shown in Fig. 1. The overall process of performing the analysis is shown in Fig. 2.

#### Data acquisition

We utilized genome-wide association study (GWAS) datasets from the MRC Integrated Epidemiology Unit (IEU) GWAS database (https://gwas.mrcieu.ac.uk/), which provides open-access summary statistics for a wide range of phenotypes. Specifically, summary-level GWAS data for spermidine levels were obtained from a UK Biobank GWAS (ebi-a-GCST90026265), as described by Panyard et al. [17], including 6,874,606 SNPs in individuals of European ancestry. For CHD, we used data from the CARDIoGRAMplusC4D consortium meta-analysis (ieu-a-8), comprising over 60,000 CHD cases and 120,000 controls of predominantly European descent, with 2,420,361 SNPs analyzed [18]. In this dataset, CHD was defined as a composite endpoint encompassing myocardial infarction, acute coronary syndrome, chronic stable angina, and significant coronary stenosis, corresponding to ICD-10 codes I20–I25 and ICD-11 codes BA41.0–BA41.Z. Both datasets focused on individuals of European ancestry to minimize population stratification bias. All GWAS summary data were accessed via the IEU OpenGWAS platform. Further details regarding sample sizes, population characteristics, and study design are provided in Table 1 and the Data Availability Statement.

**Table 1:** General information about the datasets

Phenotype	Group ID	Database	Year	Number of SNPs	Population
Spermidine level	ebi-a-GCST90026265	IEU GWAS	2021	6,874,606	European
CHD	ieu-a-8	CARDIoGRAM	2011	2,420,361	European

Spermidine level, SL; Coronary heart disease, CHD; Single nucleotide polymorphisms, SNPs; Integrated Epidemiology Unit (IEU); Genome-wide association studies, GWAS

#### Selection of genetic instrumental variables (IVs)

SNPs were selected based on genome-wide significance (*P* < 5 × 10^-8) and, when necessary, suggestive significance (*P* < 5 × 10^-5). We ensured minimal linkage disequilibrium (R^2 < 0.001 within a 10,000 kb window) [19] and excluded SNPs with potential pleiotropic effects using the PhenoScanner database [20]. The strength of the instrumental variables was verified through F-statistics and variance explained (R^2) [21]. To assess the applicability of genetic instruments across ancestries, we compared the minor allele frequencies (MAFs) of the included SNPs between European and East Asian populations using data from the 1000 Genomes Project (see Supplementary Table S3 and Text S1 for details).

#### MR statistical approach

The primary statistical method used was the inverse variance weighted (IVW) approach, which integrates SNP-specific Wald ratios via meta-analysis [22]. This method was supplemented by the weighted median, MR-Egger [23], and maximum likelihood approaches to ensure robustness [24]. The MR-Egger method also facilitated testing for horizontal pleiotropy and adjusting accordingly.

#### Sensitivity and visualization analyses

Sensitivity analyses, including Cochrane’s Q test for heterogeneity [25] and MR-Egger intercept for horizontal pleiotropy [23], were conducted. In our MR analyses, the inverse-variance weighted (IVW) method was used as the primary approach to estimate the causal effect of spermidine levels on CHD [26]. If Cochran’s Q test indicated significant heterogeneity among SNP-specific Wald ratio estimates, a random effects IVW model was employed to account for between-instrument variability [27]. In this context, the SNPs served as the random effects, reflecting possible differences in effect size due to horizontal pleiotropy or other sources of heterogeneity across genetic instruments [28]. The overall causal estimate was modeled as a fixed effect. Importantly, no traditional patient-level confounders (such as age, sex, or other clinical variables) were included as fixed or random effects, as MR analyses based on summary-level GWAS data operate at the level of genetic instruments rather than individual participants [29]. This approach follows established MR analytical guidelines to ensure robust and unbiased estimation of causal effects while appropriately addressing instrument heterogeneity. The MR-PRESSO test was used to assess the impact of outliers on the results. Graphical representations such as forest plots, funnel plots, and scatter plots were employed to summarize relationships between spermidine, CHD, and SNP effects [30].

#### Statistical execution

Analysis was conducted in the R software environment using the MR-PRESSO and TwoSampleMR packages [31]. All scripts were made available for transparency and reproducibility.

### Method development, performance verification, and clinical sample collection and analysis of mass spectrometry for the detection of spermidine concentration in plasma

#### Instrumentation and chromatographic conditions

To accurately measure plasma spermidine concentrations, we utilized a highly sensitive LC-MS/MS setup. The system comprised a Thermo Fisher triple quadrupole mass spectrometer, model TSQ Altis and equipped with an electrospray ionization source (ESI+). For chromatographic separation, we employed a Waters BEH T3 chromatographic column (100 × 2.1 mm, 1.8 μm particle size). The injection volume was set at 5µL, with column temperature maintained at 45℃, and the autosampler temperature at 4℃ to preserve sample integrity. The mobile phases used were a water solution with 0.1% formic acid (Mobile Phase A) and acetonitrile with 0.1% formic acid (Mobile Phase B), delivered at a flow rate of 0.400 mL/min. Gradient elution optimized separation, beginning with a 90:10 A: B ratio, peaking at 5:95 between 0.5 and 2 min, then returning to 90:10 until the end of the 3-minute run.

#### Mass spectrometry conditions

The mass spectrometry parameters were meticulously set to ensure high sensitivity. Ion source conditions included a collision gas pressure of 3–6 psi, a curtain gas (CUR) of 30–50 psi, and ion source gases (Gas1 and Gas2) at 50–80 psi. The spray voltage was adjusted to 3000 V, with the source temperature at 350℃ and the atomization temperature at 450℃. The sheath, auxiliary, and sweep gas flows were maintained at 45, 15, and 5 arb, respectively. Selected reaction monitoring (SRM) was conducted in positive ion mode, focusing on transitions of spermidine from a precursor ion of 146.2 m/z to product ions of 72.2 and 112.2 m/z, with collision energies of 14 V and 13 V. An internal standard, spermidine-d8, was monitored from a precursor ion of 154.2 m/z to product ions of 80.2 and 120.2 m/z, under the same collision energies.

#### Performance validation of mass spectrometry method

The LC-MS/MS method for detecting spermidine in plasma underwent rigorous performance validation in line with established analytical guidelines. Key parameters assessed included linearity, sensitivity, precision, accuracy, and recovery, ensuring a robust and reliable assay. Calibration curves were generated for a range of expected spermidine concentrations in plasma, demonstrating excellent linearity across the range. The sensitivity of the method was confirmed through the determination of the limit of detection (LOD) and the limit of quantification (LOQ), which were established at levels low enough to detect minute quantities of spermidine with high confidence. Precision and accuracy tests were conducted on 3 days, and recovery studies confirmed the method’s ability to accurately recover spermidine from plasma samples.

#### Clinical sample collection and spermidine analysis

Blood samples were collected in anticoagulation tubes, and plasma was separated by centrifugation at 4,000 rpm for 10 min. The plasma was then stored at -80 °C to preserve spermidine integrity until analysis. On the day of testing, plasma samples were thawed, and spermidine was extracted using a protein precipitation method. To extract spermidine, 150µL of methanol: acetonitrile (1:2) was added to 50µL of plasma. After precipitation, the samples were centrifuged, and the clear supernatant was collected for spermidine analysis by LC-MS/MS. Each sample was analyzed in duplicate to minimize analytical errors and ensure result repeatability. The measured spermidine concentrations were then used to assess their correlation with CHD and associated risk factors.

#### Statistical data processing

Statistical analysis to validate the method’s performance was carried out using SPSS software, version 19.0. Quantitative data followed the normal distribution expressed as mean ± standard deviation (SD), while non-normally distributed data were described as median (interquartile range). Counting data were presented as percentages.

### Patient population

This study included two main groups: 300 CHD patients and 200 normal volunteers. CHD patients were recruited from Beijing Anzhen Hospital and diagnosed based on established clinical criteria, including coronary angiography, history of myocardial infarction, or a positive stress test. The control group consisted of healthy volunteers with no known history of cardiovascular disease, hypertension, hyperglycemia, or other chronic conditions. Ethical approval for the study was obtained from the Beijing Anzhen Hospital Institutional Review Board (IRB), and written informed consent was provided by all participants.

In addition to the main CHD and control groups, the study included subgroups based on specific risk factors:

1. Hypertension subgroup: Patients were classified into those with hypertension (systolic BP ≥ 140 mmHg or diastolic BP ≥ 90 mmHg) and those with normotension (systolic BP < 120 mmHg and diastolic BP < 80 mmHg).
2. Hyperglycemia subgroups: Patients with hyperglycemia (fasting glucose ≥ 126 mg/dL or HbA1c ≥ 6.5%) were compared to normoglycemic individuals (fasting glucose < 100 mg/dL and HbA1c < 5.7%).
3. Hyperlipidemia subgroups: Hyperlipidemia was defined by elevated total cholesterol (TC ≥ 240 mg/dL), low-density lipoprotein cholesterol (LDL-C ≥ 160 mg/dL), or triglycerides (TG ≥ 200 mg/dL). Participants were classified into those with hyperlipidemia and those with normal lipid profiles.
4. Age subgroups: Participants were categorized into two age groups: those aged 65 years or older and those younger than 65 years.
5. Additional risk factors: Body mass index (BMI), waist-to-height ratio, and gender, which were also collected and analyzed for correlation with spermidine levels and CHD. Demographic data, including age, sex, BMI and medical history, were collected for all participants.

## Results

### Effect of spermidine and CHD

For the MR analysis, we selected genetic instruments (SNPs) associated with spermidine levels that met the threshold of genome-wide significance (*p* < 5e-5) and were located within a 10,000 kb window to minimize confounding. After removing variants with linkage disequilibrium (r²< 0.001) and applying a MAF filter of > 0.01, 50 SNPs were identified as valid instruments for spermidine. Potential confounders related to known CHD risk factors were excluded using the PhenoScanner tool. Details of the selected SNPs are provided in Additional File- 1 (Table S1-S2).

**Fig. 3:**
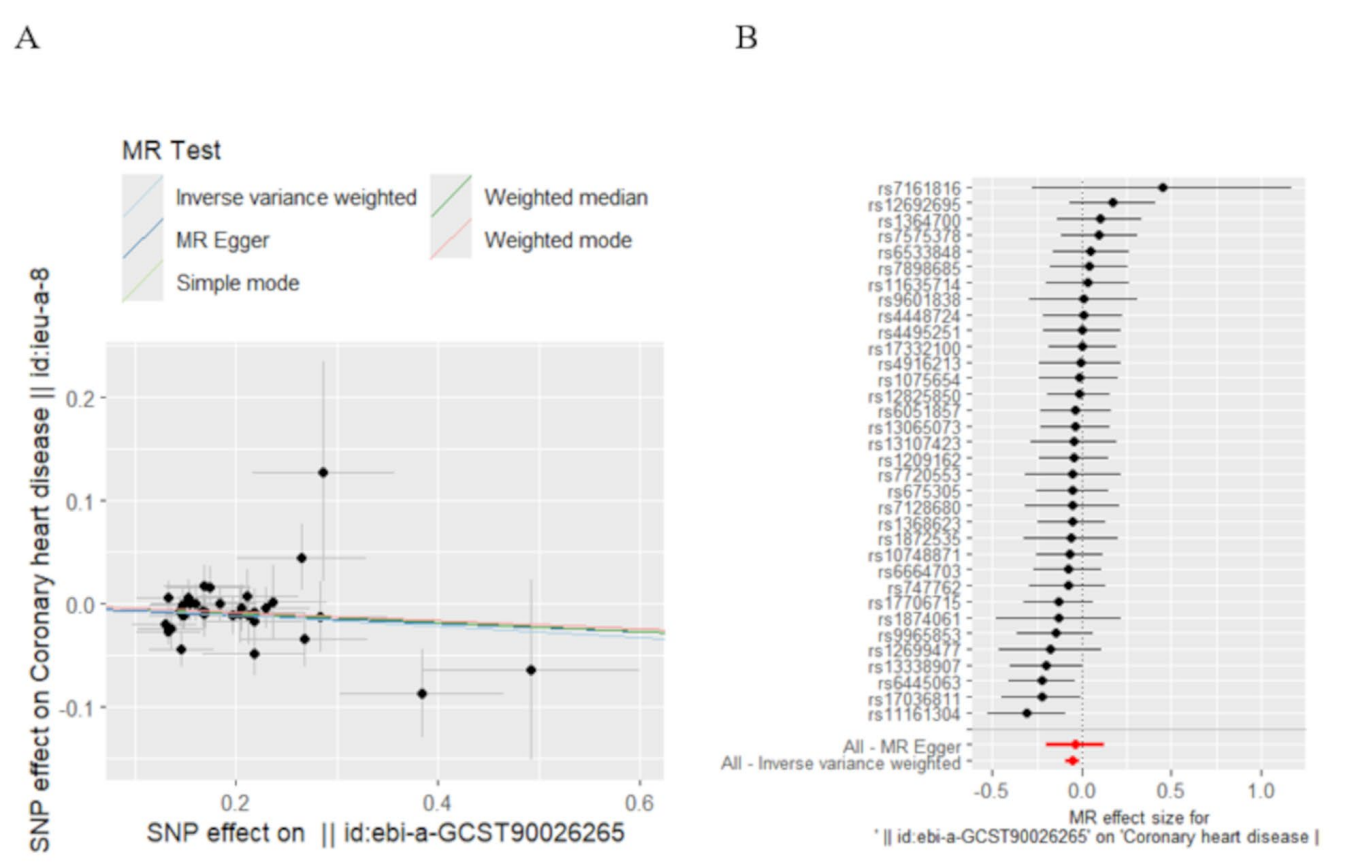
Scatter plot of MR analysis (**A**) and Forest plot (**B**) showing the causal effect of spermidine levels on CHD in initial practice. From top to bottom, the lines correspond to the weighted median, weighted mode, inverse variance weighted, simple mode, and MR Egger methods.Each black dot represents the effect estimate of a single SNP, and the horizontal line through the dot represents its 95% confidence interval. The pooled MR estimate is depicted by red line, with the length on either side indicating the whole 95% CI

**Fig. 4:**
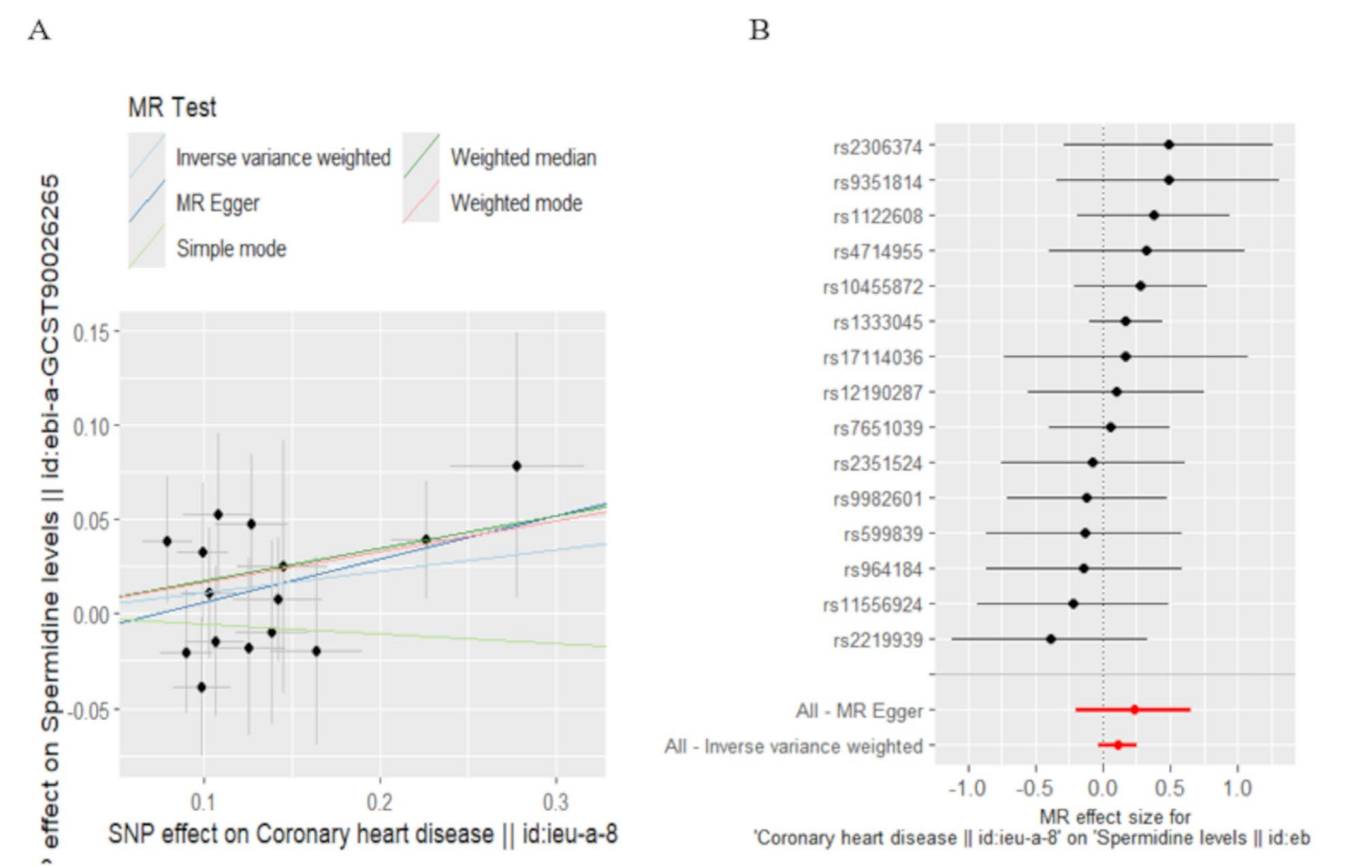
Scatter plot of MR analysis (**A**) and Forest plot (**B**) showing the causal effect of CHD on spermidine levels in initial practice. From top to bottom, the lines correspond to the weighted median, weighted mode, inverse variance weighted, MR Egger methods and simple mode. Each black dot represents the effect estimate of a single SNP, and the horizontal line through the dot represents its 95% confidence interval. The pooled MR estimate is depicted by red line, with the length on either side indicating the whole 95% CI

The MR analysis was performed using multiple methods to assess the causal effect of spermidine on CHD risk. As shown in Table 2; Figs. 3 and 4, our results indicate a significant association between lower genetically predicted spermidine levels and an increased risk of CHD. Specifically, the IVW method revealed that reduced spermidine was linked to a higher risk of CHD (*p* = 0.0046, beta = -0.054). This association remained significant after performing a maximum likelihood ratio test (*p* = 0.0057, beta=-0.054), further supporting the robustness of this finding. These results suggest that decreased spermidine levels may play a causal role in the development of CHD.

**Table 2:** Different MR methods of assessing the causal effect between SL and CHD

NO.SNPs IVW^a^				simple mode^a^			weighted median^a^			MR-Egger^a^			weighted mode^a^		
	95%CI	Beta	*p*	95%CI	Beta	*p*	95%CI	Beta	*p*	95%CI	Beta	*p*	95%CI	Beta	*p*
SL-CHD	34 0.912, 0.871,1.058	0.983	-0.054	0.0046	-0.04	0.42	0.90, 1.00	-0.04	0.08	0.81, 1.12	-0.03	0.63	0.87, 1.05	-0.04	0.39
CHD-Sl	15 0.96,1.29	0.76	0.11	0.129	0.68,1.31	-0.05	0.97,1.45	0.17	0.09	0.81,1.93	0.22	0.31	0.91,1.50	0.16	0.21

Spermidine level,SL; Coronary heart disease,CHD;p-value,p; No MR-PRESSO outliers were exposed (NA), a

In contrast, the reverse analysis showed no evidence that CHD influences spermidine levels. The IVW estimate for CHD on spermidine was not significant (*p* = 0.129), indicating that the occurrence of CHD does not appear to cause a reduction in spermidine levels.

To confirm the validity of our results, we conducted sensitivity analyses, including the MR-Egger regression and leave-one-out analysis, which did not indicate significant heterogeneity or pleiotropy among the genetic instruments used. The MR-Egger regression intercept was close to zero (intercept = − 0.0026, SE = 0.010, *p* = 0.85; Table 3), indicating no evidence of horizontal pleiotropy. In MR analyses, an intercept significantly different from zero would suggest that the genetic variants affect the outcome through pathways other than the exposure of interest, thereby biasing the causal estimate. Thus, an intercept close to zero supports the validity of the genetic instruments and the robustness of the causal inference. Additionally, the funnel plot and MR-PRESSO tests did not detect any outliers, further supporting the reliability of our findings.

**Table 3:** Heterogeneity and horizontal Pleiotropy check between SL and CHD

Exposure	outcome	Horizontal pleiotropy test (MR-Egger)			Heterogeneity test (IVW)		Heterogeneity test (MR-Egger)	
		Intercept	SE	*P*	*Q*	*p*	*Q*	*p*
SL	CHD	-0.0026	0.01	0.85	27.48	0.73	27.44	0.69
CHD	SL	-0.0016	0.0029	0.58	8.00	0.88	7.69	0.86

*p*-value,*p*;Cochran,s Q test,Q

### LC-MS/MS analysis of spermidine

#### Specificity and retention time

**Fig. 5:**
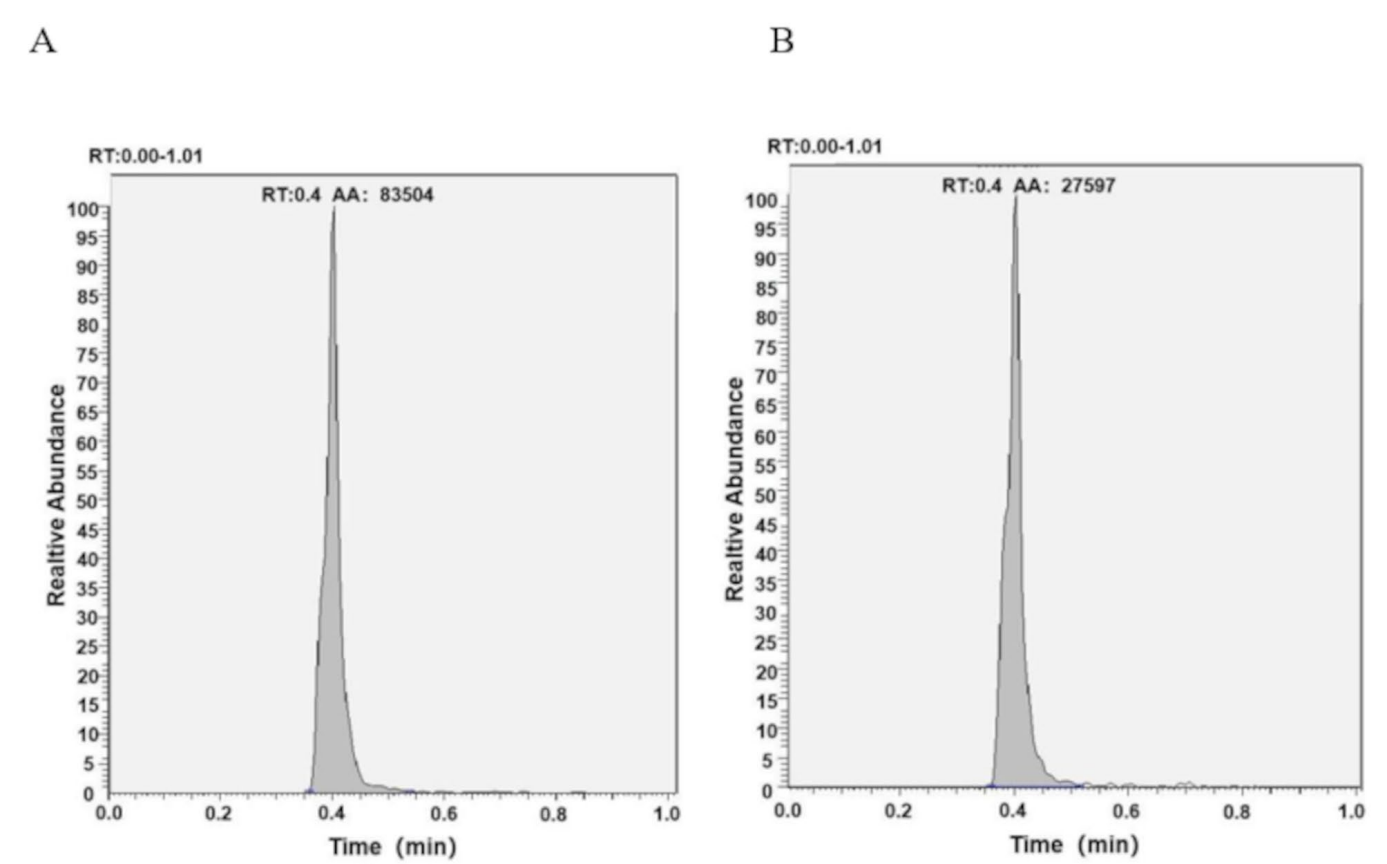
The chromatogram of spermidine (**A**) and the internal standard spermidine-d8 (**B**)

Chromatographic separation of spermidine was achieved under optimal conditions, with spermidine displaying a retention time of 0.40 min, ensuring clear separation from potential interferences. Spermidine-d8, the isotopically labeled internal standard, exhibited a retention time of 0.40 min. The high-resolution mass spectrum, which confirms the specificity of the detection, is presented in Fig. 5.

#### Calibration curve and sensitivity

The quantification of spermidine in plasma showed a strong linear response across a range of 5 to 200ng/mL. The calibration curve was defined by the equation: y = 1.566*10^-3-1.7*10^-3(R²= 0.9997), demonstrating high linearity. The lower limit of quantitation for spermidine was established at 5ng/mL, indicating excellent sensitivity.

#### Precision and accuracy

The method’s precision and accuracy were validated using quality control samples at four concentration levels (Quantification Limit, low, medium, and high). The intra-day and inter-day variations for spermidine measurements were consistently below 10%. Detailed performance data are available in Table 4.

**Table 4:** Intra-batch and Inter-batch precision and accuracy of spermidine

Name	Level	Batch 1		Batch 2		Batch 3		Inter-batch	
		Precision (%)	Accuracy (%)	Precision (%)	Accuracy (%)	Precision (%)	Accuracy (%)	Precision (%)	Accuracy (%)
**Spermidine**	LLOQ	2.25	-0.78	6.87	6.23	2.53	2.74	5.13	2.73
	QCL	1.58	1.31	3.1	-1.26	0.94	1.93	2.39	0.66
	QCM	0.96	0.91	5.37	-1.82	1.35	-0.38	3.22	-0.43
	QCH	1.88	1.05	0.79	0.04	1.44	2.05	1.59	1.05

LLOQ: Lower Limit of Quantification; QCL: Quality Control Low; QCM: Quality Control Medium; QCH: Quality Control High

#### Stability assessment

Spermidine showed high stability in plasma samples under various storage conditions. Recovery rates remained above 95% after 12 h in the autosampler, through three freeze-thaw cycles, and after 30 days at -20 °C. Relative standard deviations (RSDs) were maintained below 5%, confirming the method’s suitability for long-term studies.

#### External quality control results

Low Quality Control (LQC) results demonstrated that at a target spermidine level of 15ng/mL, the measured concentration was 15.9ng/mL, translating to a deviation of 6%n. This level reflects the method’s precision within its lower range. At a higher quality control (HQC) level, a target spermidine concentration of 150ng/mL compared to the measured value of 153.1ng/mL indicates a deviation of 2.07%, highlighting consistent accuracy across the method’s operational range.

### Clinical analysis of spermidine levels in CHD patients and controls

This section of the study examines spermidine levels across different demographic and clinical subgroups to determine associations with CHD and other risk factors. The study included 300 CHD patients and 200 normal volunteers (Table 5).

**Table 5:** Analysis of plasma spermidine concentrations across different demographic and clinical subgroups

Category	Subcategory	Count	Spermidine (ng/mL)	Statistical Method	*p*-value
**Gender**	Male	225	18.18 ± 9.28	Unpaired t-test	0.5752
	Female	74	17.51 ± 7.9		
**Age**	< 65	232	18.87 ± 9.25	Unpaired t-test	0.002
	≥ 65	67	15.05 ± 7.16		
**BMI**	< 18.5	4	16.53 ± 5.05	ANOVA	0.7573
	18.5–23.9	76	18.02 ± 8.18		
	24-27.9	143	18.49 ± 8.66		
	≥ 28	76	17.17 ± 10.19		
**Waist-to-Height**	< 0.5	11	17.31 ± 7.47	Unpaired t-test	0.7913
	≥ 0.5	288	18.04 ± 9.02		
**Hypertension**	Non-HTN	169	19.29 ± 8.72	Unpaired t-test	0.0306
	HTN	130	17.03 ± 9.01		
**Diabetes**	Non-DM	192	18.16 ± 8.92	Unpaired t-test	0.7039
	DM	107	17.75 ± 9.06		
**Hyperlipidemia**	Non-HLD	118	18.48 ± 8.99	Unpaired t-test	0.4684
	HLD	181	17.71 ± 8.94		

#### Spermidine levels in CHD patients versus normal volunteers

Analysis showed that patients with CHD had significantly lower levels of spermidine compared to normal volunteers. The mean spermidine level in CHD patients was 18.01 ± 8.95 ng/mL compared to 32.7 ± 10.84 ng/mL in the control group, indicating a reduction in spermidine levels in the CHD group (*p* < 0.05).

#### Effect of age on spermidine levels

In the age-based subgroup analysis, participants aged 65 years and older had significantly lower levels of spermidine than those younger than 65 years. The mean spermidine levels were 15.05 ± 7.16 ng/mL in the older group compared to 18.87 ± 9.25 ng/mL in the younger group (*p* < 0.05).

#### Spermidine levels and hypertension

The hypertensive subgroup demonstrated lower spermidine levels compared to the normotensive subgroup. Specifically, the mean spermidine levels were 17.03 ± 9.01 ng/mL in hypertensive patients versus 19.29 ± 8.72 ng/mL in normotensive individuals (*p* < 0.05).

#### Other comparisons

No significant differences in spermidine levels were observed when comparing hyperglycemic (17.75 ± 9.06 ng/mL) to normoglycemic individuals (18.16 ± 8.92 ng/mL), or between participants with hyperlipidemia (18.48 ± 8.99 ng/mL) and those with normal lipid profiles (17.71 ± 8.94 ng/mL). Additionally, no significant correlations were noted between spermidine levels and BMI, waist-to-height ratio, or gender.

## Discussion

Our study provides compelling evidence that lower genetically predicted levels of spermidine are associated with an increased risk of CHD. To ensure the robustness of our causal inference, we applied several MR methods—including IVW, MR-Egger, weighted median, simple mode, and weighted mode. The IVW method revealed a significant inverse association between genetically predicted spermidine levels and CHD risk, and this was supported by consistent effect directions from the weighted median, MR-Egger, and mode-based estimators. While only the IVW and maximum likelihood models reached statistical significance, all methods pointed to a similar protective effect of spermidine. Importantly, MR-Egger analysis showed no evidence of horizontal pleiotropy, supporting the validity of our findings. Taken together, the convergence of results across different MR methods increases confidence that lower spermidine levels may play a causal role in the development of CHD. This finding is notable, as spermidine is well established for its roles in promoting cellular autophagy, modulating inflammatory pathways, and supporting endothelial function [1, 32]. In biological terms, reduced spermidine levels may predispose individuals to CHD through several interrelated mechanisms. Spermidine is a potent inducer of autophagy, facilitating the removal of damaged proteins and organelles and thereby reducing oxidative stress within vascular tissues. Deficiency in spermidine may impair this protective clearance, leading to excess reactive oxygen species and mitochondrial dysfunction [33]. In addition, spermidine exerts anti-inflammatory effects by modulating macrophage activation and downregulating pro-inflammatory cytokines, processes that are central to atherosclerotic plaque formation [34]. Spermidine also helps maintain endothelial health by enhancing nitric oxide bioavailability and protecting against endothelial dysfunction, a pivotal early event in CHD progression [35]. Taken together, these observations suggest that spermidine acts not merely as a biomarker, but also as a biological mediator with preventative potential in the pathogenesis of CHD. These biological processes are particularly relevant to CHD, which is characterized by clinical manifestations of myocardial ischemia, such as angina pectoris and myocardial infarction, resulting from chronic atherosclerosis and acute vascular events. Spermidine’s ability to facilitate the removal of cellular debris, limit oxidative stress, and preserve endothelial health may thus contribute to reducing both the incidence and severity of clinical CHD events [1].

By comparing our results to previous research, we find that our study not only reinforces the cardioprotective effects of spermidine observed in epidemiological and experimental settings, but also advances the field by establishing a likely causal link between lower spermidine levels and the risk of CHD, using a bidirectional MR framework. This methodology offers a substantial advantage over traditional observational studies by minimizing confounding and clarifying the directionality of the association. Importantly, our data suggest that CHD itself does not lead to a significant reduction in spermidine levels, indicating that reduced spermidine is more likely a predisposing factor rather than a consequence of overt CHD. This highlights the value of spermidine as a predictive biomarker for CHD risk rather than as a marker of disease progression.

Our subgroup analyses reveal important patterns in spermidine distribution within the CHD patient cohort. Notably, lower spermidine levels are more frequently observed among older adults and those with hypertension, both of whom represent high-risk populations for CHD-related events. This suggests that age-related declines in autophagic activity and increased oxidative stress may amplify susceptibility to CHD when spermidine is deficient. Furthermore, hypertension-associated vascular injury may be partially mitigated by higher spermidine concentrations, which is consistent with our observation of lower spermidine in hypertensive CHD patients. Hypertension is well recognized to accelerate vascular damage through chronic hemodynamic stress, oxidative stress, and promotion of endothelial dysfunction [36]. Under sustained high pressure, endothelial cells exhibit impaired nitric oxide bioavailability, increased reactive oxygen species generation, and heightened inflammatory activation, all of which facilitate vascular remodeling and atherogenesis [37]. Spermidine may counteract these deleterious processes by enhancing autophagic clearance of damaged cellular components, reducing oxidative stress, and preserving endothelial nitric oxide synthase (eNOS) activity [1]. By maintaining endothelial integrity and limiting hypertensive vascular injury, spermidine helps protect against the downstream progression of coronary atherosclerosis [38]. Thus, the reduced spermidine levels we observed in hypertensive individuals may represent a diminished protective buffer against endothelial dysfunction, providing mechanistic support for their increased vulnerability to CHD. In contrast, we did not observe significant differences in spermidine levels based on glycemic or lipid status, implying that the relationship between spermidine and CHD risk is less directly influenced by hyperglycemia or dyslipidemia.

These observations support a potential targeted prevention strategy—especially in elderly and hypertensive individuals—where increasing spermidine intake or endogenous levels could reduce CHD risk or delay disease onset. Mechanistically, spermidine’s promotion of autophagy, anti-inflammatory effects, and enhancement of endothelial nitric oxide production [39, 40] collectively underpin its protective role in the pathogenesis of CHD. It is also worth noting that the effect of spermidine may vary across different demographic and clinical groups; for example, younger individuals or those without hypertension may not derive the same magnitude of benefit, likely due to higher baseline levels of autophagic activity and vascular function.

Our results thus highlight the importance of considering patient heterogeneity and risk profiles in the application of metabolic or nutritional interventions for CHD. The use of a validated LC-MS/MS method to quantify plasma spermidine further demonstrates the practical feasibility of measuring this metabolite in clinical settings, enhancing the translational value of our findings. Collectively, our study underscores the potential for spermidine not only as a biomarker for CHD risk assessment, but also as a promising candidate for future interventional trials aiming to prevent or mitigate clinical CHD events through dietary or pharmacological supplementation.

Despite these insights, our study has limitations. The potential for residual confounding and biases inherent in any observational study [41], even those using MR, and the assumption that the genetic instruments affect the outcome only through spermidine levels, cannot be entirely excluded. A further methodological consideration is the use of statistical fine-mapping approaches, such as the Sum of Single Effects (SuSIE) model, to more precisely identify causal variants at GWAS loci. Fine-mapping can improve the specificity of Mendelian randomization analyses by helping to select variants most likely to have a direct biological effect, thereby reducing the potential influence of non-causal or weakly associated SNPs. However, we did not employ SuSIE or similar methods in the present study due to several factors. First, our analyses were based on publicly available summary-level GWAS data, which lack the individual-level genotypes and dense regional coverage needed for optimal fine-mapping. Second, the accuracy of fine-mapping depends on access to appropriate, high-resolution linkage disequilibrium reference panels that match the study population. Finally, statistical fine-mapping approaches have their own limitations, including sensitivity to LD structure, potential model misspecification, and challenges in resolving multiple causal variants in complex regions. Future studies using individual-level data, high-density genotyping, and robust LD reference resources will be better positioned to integrate these advanced techniques and further strengthen causal inference in MR analyses. Additionally, a potential limitation of our study is the lack of formal colocalization analysis to confirm whether the same causal variant is responsible for both spermidine levels and CHD risk at each locus. Due to data constraints and the limited availability of high-resolution locus-specific summary statistics for spermidine, we were unable to perform robust colocalization testing. Future studies leveraging larger GWAS datasets and individual-level data will be necessary to address this important question. A key limitation of our study is that the MR analysis used GWAS data of European ancestry, while the clinical cohort consisted of Asian individuals. To address this, we compared the minor allele frequencies of instrumental SNPs using data from the 1000 Genomes Project and found general concordance between European and East Asian populations for most SNPs. However, differences in linkage disequilibrium and potential gene-environment interactions may affect the generalizability of the MR results. Future studies using GWAS data from Asian populations are warranted, as is the need for standardization of LC-MS/MS methods between different laboratories, highlighting areas for future research. Furthermore, unmeasured confounders may exist due to differences in dietary patterns, lifestyle, and socioeconomic factors between European and Asian populations. Of note, Asian populations have lower intakes of spermidine-rich foods, such as milk and oats, partly due to higher rates of lactose intolerance. These differences may influence baseline spermidine levels and their association with coronary artery disease risk. Although the clinical cohort analysis was adjusted for major cardiovascular risk factors, formal food frequency questionnaires or socioeconomic surveys were not conducted. This limited our ability to directly assess or adjust for these potential confounders, and the generalizability of the European MR analysis to Asian populations should be interpreted with caution. Future studies that include detailed dietary and socioeconomic data are necessary to address these limitations. Addressing these limitations through longitudinal studies, exploring the relationships between spermidine and other cardiovascular risk factors, and conducting interventional trials will enhance our understanding of spermidine’s role in cardiovascular health and its potential as a target for therapeutic interventions. In particular, a randomized controlled trial of spermidine supplementation would be a reasonable and necessary next step to directly evaluate its effects on CHD progression and clinical outcomes. Such a trial could help determine whether the protective associations we observed translate into therapeutic benefit in at-risk populations.

In summary, our study contributes novel evidence for a causal relationship between lower spermidine levels and increased risk of coronary heart disease, offering a new perspective on metabolic interventions for CHD prevention, particularly in aging and hypertensive populations.

## Conclusion

This study provides evidence that lower levels of spermidine are associated with an increased risk of CHD. By leveraging a bidirectional MR approach, our results support a likely causal relationship, positioning reduced spermidine as a potential precursor to CHD, with implications for its use as a preventative biomarker, particularly in high-risk groups like older adults and hypertensive individuals. Future research should extend these findings across diverse populations, explore longitudinal relationships, and assess the therapeutic potential of spermidine supplementation to fully harness its benefits in cardiovascular disease prevention and management.

## Supplementary Information

Below is the link to the electronic supplementary material.

Supplementary Material 1

Supplementary Material 2

## Data Availability

No datasets were generated or analysed during the current study.

## Declarations

### Clinical trial number

Beijing Anzhen Hospital Ethics Committee Approval Number: KS2024131.
