# Supplementary material for: Analysis of spermidine’s effect on coronary heart disease risk using bidirectional Mendelian randomization and LC-MS/MS
Source: Hereditas. 2025 Sep 26;162:189. doi: 10.1186/s41065-025-00568-4 (PMC12465617; doi:10.1186/s41065-025-00568-4)
Supplement: Supplementary file 1 — Supplementary Material 1 [file 41065_2025_568_MOESM1_ESM.docx]

Table S1. Included SNPs that are dramatically associated with Spermidine levels.

| **NO.** | **SNPs** | **POS** | **Chr** | **β** | **SE** | **Effect-allele** | **Other-allele** | ***p*** |
| --- | --- | --- | --- | --- | --- | --- | --- | --- |
| 1 | rs150584441 | 111867940 | 1 | -0.7627 | 0.172 | T | C | 9.26105E-06 |
| 2 | rs6694576 | 236977509 | 1 | -0.1418 | 0.0331 | A | G | 1.85102E-05 |
| 3 | rs190032530 | 44994051 | 1 | -0.6102 | 0.1481 | T | C | 3.79804E-05 |
| 4 | rs4916213 | 173268821 | 1 | -0.1467 | 0.0327 | C | T | 0.000007193 |
| 5 | rs284059 | 188447875 | 1 | -0.5021 | 0.1213 | A | G | 3.48803E-05 |
| 6 | rs6664703 | 150459087 | 1 | -0.1468 | 0.0327 | C | G | 7.38006E-06 |
| 7 | rs78473397 | 184872450 | 1 | -0.3368 | 0.0757 | T | C | 8.60201E-06 |
| 8 | rs77551695 | 230350848 | 1 | -0.3463 | 0.0783 | G | C | 9.86393E-06 |
| 9 | rs77926168 | 87500215 | 1 | -0.4425 | 0.1036 | G | A | 1.93798E-05 |
| 10 | rs12117600 | 247741618 | 1 | 0.1444 | 0.0355 | C | T | 4.81105E-05 |
| 11 | rs76168185 | 58808704 | 1 | -0.5086 | 0.1243 | T | A | 0.00004314 |
| 12 | rs66794250 | 3773332 | 2 | -0.1757 | 0.0424 | A | G | 3.37598E-05 |
| 13 | rs12692695 | 164440010 | 2 | -0.2648 | 0.064 | G | T | 0.00003535 |
| 14 | rs1364700 | 220697472 | 2 | -0.1691 | 0.0398 | T | G | 0.00002121 |
| 15 | rs17036811 | 47902698 | 2 | -0.3847 | 0.0808 | C | T | 1.90099E-06 |
| 16 | rs149233695 | 44839083 | 2 | -0.4576 | 0.1103 | T | G | 3.32598E-05 |
| 17 | rs7575378 | 197500771 | 2 | 0.1735 | 0.042 | T | C | 3.54397E-05 |
| 18 | rs115922453 | 66908476 | 2 | -0.4105 | 0.0991 | C | T | 3.46601E-05 |
| 19 | rs6445063 | 172193632 | 3 | -0.2176 | 0.0509 | C | T | 1.90401E-05 |
| 20 | rs146372531 | 61308149 | 3 | -0.4162 | 0.0919 | A | G | 5.91602E-06 |
| 21 | rs9829165 | 187917628 | 3 | -0.5486 | 0.1242 | A | G | 9.94901E-06 |
| 22 | rs72979373 | 128148172 | 3 | 0.3 | 0.0708 | T | C | 2.24999E-05 |
| 23 | rs13065073 | 107448644 | 3 | 0.1674 | 0.0391 | G | A | 1.81999E-05 |
| 24 | rs78379154 | 96613214 | 3 | -0.5664 | 0.1251 | G | T | 5.97998E-06 |
| 25 | rs76521787 | 45631492 | 4 | -0.5859 | 0.1298 | T | C | 6.40398E-06 |
| 26 | rs138127319 | 188200369 | 4 | -0.7342 | 0.1693 | T | C | 1.44501E-05 |
| 27 | rs6533848 | 116228827 | 4 | -0.1332 | 0.0311 | G | T | 1.78599E-05 |
| 28 | rs13107423 | 58112657 | 4 | -0.2827 | 0.0669 | T | C | 2.41702E-05 |
| 29 | rs114007855 | 7735585 | 4 | -0.5362 | 0.1313 | T | C | 4.41998E-05 |
| 30 | rs141354610 | 21173754 | 4 | -0.3246 | 0.0612 | G | A | 1.12001E-07 |
| 31 | rs55994030 | 165733471 | 5 | -0.2307 | 0.0536 | G | A | 1.64801E-05 |
| 32 | rs147497213 | 154162978 | 5 | -0.5209 | 0.1268 | T | C | 0.00004 |
| 33 | rs7720553 | 179623637 | 5 | -0.2046 | 0.0496 | A | G | 3.67502E-05 |
| 34 | rs1874061 | 2885685 | 5 | -0.4914 | 0.1078 | C | T | 0.000005165 |
| 35 | rs17706715 | 141945931 | 5 | -0.2677 | 0.0625 | A | G | 1.81401E-05 |
| 36 | rs2548565 | 39396114 | 5 | 0.3675 | 0.0874 | A | G | 2.62102E-05 |
| 37 | rs17332100 | 9664794 | 5 | -0.1838 | 0.0376 | C | T | 1.02499E-06 |
| 38 | rs12663524 | 91600685 | 6 | -0.3753 | 0.0905 | C | G | 3.34503E-05 |
| 39 | rs112758916 | 106242055 | 6 | -0.19 | 0.0426 | T | A | 8.34104E-06 |
| 40 | rs77704179 | 36771570 | 6 | -0.2758 | 0.0675 | G | A | 4.44304E-05 |
| 41 | rs7740263 | 133366799 | 6 | -0.1397 | 0.0311 | A | G | 0.000007172 |
| 42 | rs6925329 | 166682992 | 6 | -0.1485 | 0.0344 | G | A | 1.59001E-05 |
| 43 | rs9296391 | 12475483 | 6 | -0.2347 | 0.0457 | A | G | 2.84898E-07 |
| 44 | rs4495251 | 91845703 | 6 | 0.1601 | 0.0372 | A | C | 1.67699E-05 |
| 45 | rs116650349 | 119390715 | 6 | -0.5182 | 0.1168 | T | C | 9.10207E-06 |
| 46 | rs80160659 | 85897738 | 7 | -0.226 | 0.0553 | A | G | 4.39097E-05 |
| 47 | rs10224768 | 129648273 | 7 | 0.1623 | 0.0388 | G | A | 2.89401E-05 |
| 48 | rs74868622 | 19933291 | 7 | -0.7538 | 0.1688 | G | C | 7.92903E-06 |
| 49 | rs79339942 | 156784466 | 7 | -0.2448 | 0.0598 | G | A | 4.25696E-05 |
| 50 | rs55892636 | 157867567 | 7 | -0.2178 | 0.046 | T | C | 2.21101E-06 |
| 51 | rs73737382 | 155679057 | 7 | -0.1661 | 0.0391 | G | A | 0.0000217 |
| 52 | rs12699477 | 1968953 | 7 | -0.1364 | 0.0325 | C | T | 2.77703E-05 |
| 53 | rs10095557 | 34005770 | 8 | -0.463 | 0.1046 | G | A | 9.54905E-06 |
| 54 | rs139089869 | 59675117 | 8 | -0.6883 | 0.1469 | T | G | 2.78901E-06 |
| 55 | rs62523473 | 127274995 | 8 | -0.3656 | 0.0886 | T | C | 3.69598E-05 |
| 56 | rs62489525 | 9805993 | 8 | -0.2167 | 0.0507 | G | T | 1.89099E-05 |
| 57 | rs117932370 | 112957121 | 9 | -0.5104 | 0.1249 | A | G | 0.00004346 |
| 58 | rs144889237 | 37009564 | 9 | -0.7156 | 0.1634 | T | C | 1.19201E-05 |
| 59 | rs1075654 | 136928276 | 9 | -0.2058 | 0.0499 | A | G | 3.77398E-05 |
| 60 | rs10465066 | 30337556 | 9 | -0.1496 | 0.0341 | T | G | 0.00001123 |
| 61 | rs7898685 | 22299503 | 10 | 0.1535 | 0.0353 | C | A | 1.33199E-05 |
| 62 | rs10748871 | 83790407 | 10 | -0.1486 | 0.0339 | A | G | 1.17201E-05 |
| 63 | rs4980315 | 125442588 | 10 | -0.3544 | 0.0798 | T | G | 8.99208E-06 |
| 64 | rs117918491 | 108137531 | 10 | -0.6425 | 0.1439 | C | A | 8.02509E-06 |
| 65 | rs77985321 | 14953974 | 10 | -0.2736 | 0.065 | T | C | 2.56702E-05 |
| 66 | rs7898019 | 1020204 | 10 | 0.1418 | 0.0326 | T | C | 1.32999E-05 |
| 67 | rs76501004 | 6056807 | 11 | -0.348 | 0.0816 | G | T | 2.00401E-05 |
| 68 | rs139490069 | 47983248 | 11 | -0.6964 | 0.1634 | T | C | 2.02199E-05 |
| 69 | rs7111759 | 106665159 | 11 | 0.4065 | 0.0863 | T | C | 2.49402E-06 |
| 70 | rs7113033 | 127073527 | 11 | 0.1623 | 0.0337 | A | G | 1.42801E-06 |
| 71 | rs118187827 | 87116667 | 11 | -0.4163 | 0.0987 | A | G | 2.47201E-05 |
| 72 | rs7128680 | 24338796 | 11 | -0.2128 | 0.0474 | T | C | 7.25705E-06 |
| 73 | rs12819110 | 64470011 | 12 | -0.5059 | 0.1141 | T | G | 9.21107E-06 |
| 74 | rs1534388 | 131407537 | 12 | -0.1404 | 0.0324 | C | T | 1.43999E-05 |
| 75 | rs1872535 | 128771021 | 12 | -0.1681 | 0.0373 | A | G | 6.53401E-06 |
| 76 | rs12825850 | 98249840 | 12 | -0.2298 | 0.0441 | C | T | 1.89099E-07 |
| 77 | rs4448724 | 27731160 | 12 | -0.1541 | 0.0374 | A | G | 3.70502E-05 |
| 78 | rs9601838 | 82692288 | 13 | -0.2362 | 0.0548 | T | C | 1.66001E-05 |
| 79 | rs118068566 | 19734012 | 13 | -0.5996 | 0.1311 | T | C | 4.81504E-06 |
| 80 | rs9572536 | 71293311 | 13 | -0.3747 | 0.0862 | G | C | 1.38599E-05 |
| 81 | rs112570865 | 25034673 | 13 | -0.4642 | 0.1081 | A | G | 0.00001749 |
| 82 | rs675305 | 51322188 | 13 | -0.1445 | 0.0354 | A | G | 4.40301E-05 |
| 83 | rs111484163 | 93347668 | 14 | -0.491 | 0.1161 | A | G | 0.00002326 |
| 84 | rs1209162 | 100481808 | 14 | -0.1706 | 0.0399 | G | A | 0.00001874 |
| 85 | rs113653607 | 77641605 | 14 | -0.4657 | 0.1076 | A | T | 1.49899E-05 |
| 86 | rs1368623 | 87512812 | 15 | -0.1972 | 0.044 | G | T | 7.38006E-06 |
| 87 | rs11161304 | 26636672 | 15 | -0.1462 | 0.0323 | G | A | 6.12999E-06 |
| 88 | rs74390061 | 83984884 | 15 | -0.4162 | 0.0831 | G | A | 5.424E-07 |
| 89 | rs11635714 | 101202587 | 15 | -0.2117 | 0.051 | G | T | 3.31497E-05 |
| 90 | rs7161816 | 70457246 | 15 | -0.2869 | 0.07 | G | A | 0.0000414 |
| 91 | rs72794126 | 52318025 | 16 | -0.281 | 0.0668 | T | C | 2.60699E-05 |
| 92 | rs28578193 | 67914236 | 16 | -0.8004 | 0.1825 | A | G | 1.15401E-05 |
| 93 | rs13338907 | 76007728 | 16 | 0.1333 | 0.0317 | G | A | 2.58399E-05 |
| 94 | rs747762 | 49445957 | 16 | -0.2184 | 0.0484 | T | C | 6.52304E-06 |
| 95 | rs80193311 | 8539666 | 16 | -0.3281 | 0.0766 | T | A | 1.86402E-05 |
| 96 | rs142681304 | 18112422 | 16 | -0.5743 | 0.1072 | G | C | 8.51707E-08 |
| 97 | rs11641300 | 1493151 | 16 | -0.1341 | 0.033 | T | C | 0.00004923 |
| 98 | rs112013739 | 30137351 | 17 | -0.3892 | 0.0943 | T | C | 3.66303E-05 |
| 99 | rs34662528 | 1091399 | 17 | -0.4793 | 0.1175 | T | C | 0.00004559 |
| 100 | rs2531891 | 26173508 | 17 | 0.1473 | 0.0337 | C | T | 1.23299E-05 |
| 101 | rs9965853 | 6402126 | 18 | -0.1297 | 0.032 | T | C | 4.95405E-05 |
| 102 | rs56241614 | 43879327 | 18 | 0.1437 | 0.0327 | T | C | 1.13899E-05 |
| 103 | rs117503462 | 12365119 | 18 | -0.5703 | 0.131 | T | A | 1.33199E-05 |
| 104 | rs78371360 | 9354576 | 19 | -0.334 | 0.0752 | T | C | 8.96809E-06 |
| 105 | rs6051857 | 3412564 | 20 | 0.2183 | 0.0492 | A | G | 8.96107E-06 |
| 106 | rs1028367 | 8735362 | 20 | 0.1343 | 0.0328 | G | A | 4.18302E-05 |
| 107 | rs71314897 | 111867940 | 22 | -0.278 | 0.0651 | A | G | 1.92398E-05 |

SNPs,single-nucleotide polymorphism; β,the effect size of spermidine levels; chromosome, Chr; standard error, SE; *p*, *p*-value. We used the Pheno-Scanner tool to exclude no SNPs that were related with potential pleiotropic effects. No SNP was removed for being palindromic with intermediate allele frequencies by the Two-sample MR function of the R package.

| **NO.** | **SNPs** | **POS** | **Chr** | **β** | **SE** | **Effect-allele** | **Other-allele** | ***p*** |
| --- | --- | --- | --- | --- | --- | --- | --- | --- |
| 1 | rs599839 | 109822166 | 1 | 0.106715 | 0.0169259 | A | G | 2.89001E-10 |
| 2 | rs17114036 | 56962821 | 1 | -0.144965 | 0.0255686 | G | A | 1.43001E-08 |
| 3 | rs2351524 | 203880992 | 2 | -0.13869 | 0.0206229 | C | T | 1.75995E-11 |
| 4 | rs7651039 | 15648004 | 3 | 0.142119 | 0.0252631 | C | T | 1.84999E-08 |
| 5 | rs2306374 | 138119952 | 3 | 0.108439 | 0.019636 | C | T | 3.34003E-08 |
| 6 | rs10455872 | 161010118 | 6 | 0.27787 | 0.038112 | G | A | 3.08035E-13 |
| 7 | rs9351814 | 72193707 | 6 | -0.0795946 | 0.0141883 | C | A | 2.01999E-08 |
| 8 | rs4714955 | 12903435 | 6 | -0.0997862 | 0.0145058 | T | C | 6.02976E-12 |
| 9 | rs12190287 | 134214525 | 6 | -0.103209 | 0.0156807 | G | C | 4.63981E-11 |
| 10 | rs11556924 | 129663496 | 7 | -0.0905122 | 0.0151329 | T | C | 2.21998E-09 |
| 11 | rs1333045 | 22119195 | 9 | 0.226084 | 0.019183 | C | T | 4.6302E-32 |
| 12 | rs964184 | 116648917 | 11 | -0.12596 | 0.0204991 | C | G | 8.01992E-10 |
| 13 | rs2219939 | 79029723 | 15 | -0.0990217 | 0.016288 | A | G | 1.21001E-09 |
| 14 | rs1122608 | 11163601 | 19 | -0.127428 | 0.0208429 | T | G | 9.72994E-10 |
| 15 | rs9982601 | 35599128 | 21 | 0.163991 | 0.0262564 | T | C | 4.21998E-10 |

Table S2. Included SNPs that are dramatically associated with coronary heart disease.

SNPs, single-nucleotide polymorphism;β,the effect size of coronary heart disease; chromosome, Chr; standard error, SE; p, p-value;We used the Pheno-Scanner tool to exclude no SNPs that were related with potential pleiotropic effects. No SNP was removed for being palindromic with intermediate allele frequencies by the Two-sample MR function of the R package.

Text S1. Supplementary Methods: Cross-Ancestry Allele Frequency Comparison

To evaluate the applicability of the Mendelian randomization (MR) findings across populations, we compared the minor allele frequencies (MAFs) of the instrumental single-nucleotide polymorphisms (SNPs) used in the MR analysis between European (EUR) and East Asian (EAS) populations. SNPs were selected based on their genome-wide significant associations with spermidine levels and coronary artery disease (CAD) risk from large-scale GWAS datasets.

We obtained MAF data for each SNP in both populations from the 1000 Genomes Project Phase 3 dataset ^[1]^. The EUR superpopulation includes individuals of European ancestry, while the EAS superpopulation represents East Asian ancestry. For each SNP, the effect allele, other allele, chromosome, position (hg19/GRCh37), and the nearest gene annotation were recorded. The allele frequencies were directly extracted from the “Allele frequency” section for each SNP within the respective databases.

A summary table (Supplementary Table S3) was generated, presenting side-by-side MAFs for all instrumental SNPs in both populations, along with relevant genomic information and gene annotations. This approach allows a direct visual assessment of the concordance of allele frequencies between EUR and EAS populations and facilitates interpretation of the cross-ancestry relevance of the MR findings.

Table S3. Comparison of Minor Allele Frequencies (MAFs) for Instrumental SNPs Used in Mendelian Randomization Analysis: European (EUR) vs. East Asian (EAS) Populations (1000 Genomes Project)

| SNP ID | Chr | Position | Effect Allele | Other Allele | MAF (EUR) | MAF (EAS) | Nearest Gene/Region |
| --- | --- | --- | --- | --- | --- | --- | --- |
| rs599839 | 1 | 109822166 | A | G | 0.21 | 0.08 | PSRC1 |
| rs17114036 | 1 | 56962821 | G | A | 0.4 | 0.12 | MIA3 |
| rs2351524 | 2 | 203880992 | C | T | 0.27 | 0.09 | WDR12 |
| rs7651039 | 3 | 15648004 | C | T | 0.16 | 0.36 | CNTN4 |
| rs2306374 | 3 | 138119952 | C | T | 0.17 | 0.02 | PDZRN3 |
| rs10455872 | 6 | 161010118 | G | A | 0.07 | <0.01 | LPA |
| rs9351814 | 6 | 72193707 | C | A | 0.32 | 0.39 | HLA-DQB1 |
| rs4714955 | 6 | 12903435 | T | C | 0.36 | 0.4 | TFEB |
| rs12190287 | 6 | 134214525 | G | C | 0.39 | 0.31 | CDKN1A |
| rs11556924 | 7 | 129663496 | T | C | 0.23 | 0.36 | ZC3HC1 |
| rs1333045 | 9 | 22119195 | C | T | 0.51 | 0.51 | CDKN2B-AS1 |
| rs964184 | 11 | 116648917 | C | G | 0.12 | 0.23 | ZPR1/APOA5 |
| rs2219939 | 15 | 79029723 | A | G | 0.36 | 0.38 | MAP2K5 |
| rs1122608 | 19 | 11163601 | T | G | 0.34 | 0.12 | LDLR |
| rs9982601 | 21 | 35599128 | T | C | 0.1 | 0.07 | CDKN1C |

NOTE: EUR, European ancestry; EAS, East Asian ancestry; MAF, minor allele frequency. Data are from the 1000 Genomes Project Phase 3 (http://www.internationalgenome.org/).

**Reference**

[1] 1000 Genomes Project Consortium, Auton A, Brooks LD, et al. A global reference for human genetic variation. Nature. 2015;526(7571):68-74. doi:10.1038/nature15393
