# Supplementary material for: Analysis of spermidine’s effect on coronary heart disease risk using bidirectional Mendelian randomization and LC-MS/MS
Source: Hereditas. 2025 Sep 26;162:189. doi: 10.1186/s41065-025-00568-4 (PMC12465617; doi:10.1186/s41065-025-00568-4)
Supplement: Supplementary file 2 — Supplementary Material 2 [file 41065_2025_568_MOESM2_ESM.docx]

A B


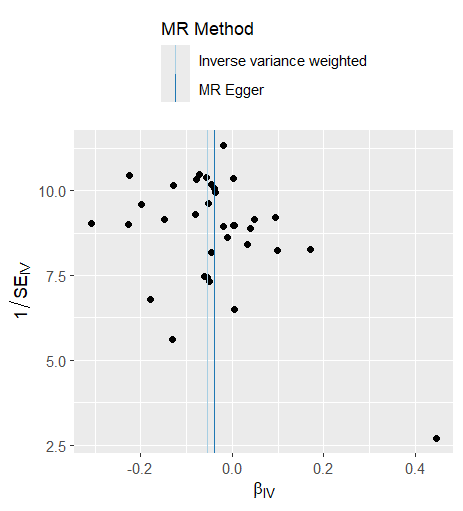

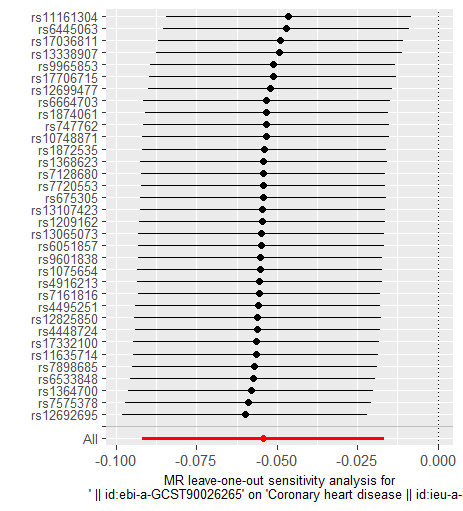


Figure1.

1. Funnel plot illustrating the MR analysis of the causal relationship between Spermidine levels and CAD, assessing the symmetry of the data;
2. Leave-one-out analysis plot depicting the causal association between Spermidine levels and CAD in the MR study.

A B


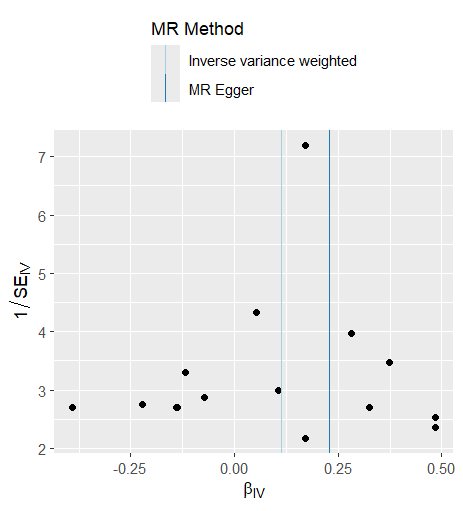

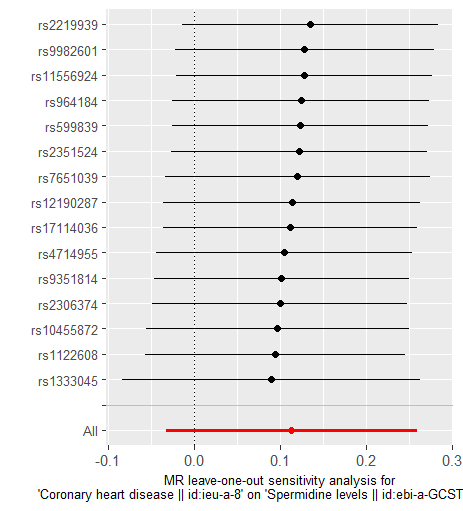


Figure 2.

A.Funnel plot illustrating the MR analysis of the causal relationship between CAD and Spermidine levels, assessing the symmetry of the data;

B.Leave-one-out analysis plot depicting the causal association between CAD and Spermidine levels in the MR study.
